# Inverse Agonist and Pharmacochaperone Properties of MK-0524 on the Prostanoid DP1 Receptor

**DOI:** 10.1371/journal.pone.0065767

**Published:** 2013-06-10

**Authors:** Pascale Labrecque, Sébastien J. Roy, Louis Fréchette, Christian Iorio-Morin, Maxime A. Gallant, Jean-Luc Parent

**Affiliations:** 1 Département de Médecine, Université de Sherbrooke, Sherbrooke, Quebec, Canada; 2 Centre de Recherche Clinique Étienne-Le Bel, Sherbrooke, Quebec, Canada; 3 Institut de Pharmacologie de Sherbrooke, Sherbrooke, Quebec, Canada; University of São Paulo, Brazil

## Abstract

Prostaglandin D_2_ (PGD_2_) acts through two G protein-coupled receptors (GPCRs), the prostanoid DP receptor and CRTH2 also known as DP1 and DP2, respectively. Several previously characterized GPCR antagonists are now classified as inverse agonists and a number of GPCR ligands are known to display pharmacochaperone activity towards a given receptor. Here, we demonstrate that a DP1 specific antagonist, MK-0524 (also known as laropiprant), decreased basal levels of intracellular cAMP produced by DP1, a Gα_s_-coupled receptor, in HEK293 cells. This reduction in cAMP levels was not altered by pertussis toxin treatment, indicating that MK-0524 did not induce coupling of DP1 to Gα_i/o_ proteins and that this ligand is a DP1 inverse agonist. Basal ERK1/2 activation by DP1 was not modulated by MK-0524. Interestingly, treatment of HEK293 cells expressing Flag-tagged DP1 with MK-0524 promoted DP1 cell surface expression time-dependently to reach a maximum increase of 50% compared to control after 24 h. In contrast, PGD_2_ induced the internalization of 75% of cell surface DP1 after the same time of stimulation. The increase in DP1 cell surface targeting by MK-0524 was inhibited by Brefeldin A, an inhibitor of transport from the endoplasmic reticulum-Golgi to the plasma membrane. Confocal microscopy confirmed that a large population of DP1 remained trapped intracellularly and co-localized with calnexin, an endoplasmic reticulum marker. Redistribution of DP1 from intracellular compartments to the plasma membrane was observed following treatment with MK-0524 for 24 h. Furthermore, MK-0524 promoted the interaction between DP1 and the ANKRD13C protein, which we showed previously to display chaperone-like effects towards the receptor. We thus report that MK-0524 is an inverse agonist and a pharmacochaperone of DP1. Our findings may have important implications during therapeutic treatments with MK-0524 and for the development of new molecules targeting DP1.

## Introduction

Prostaglandin D_2_ (PGD_2_) is a lipid mediator synthesized from arachidonic acid that directly activates two specific G protein-coupled receptors (GPCRs), the D-type prostanoid (DP) receptor [1] and chemoattractant receptor homologous molecule expressed on T-helper type 2 cells (CRTH2) [2], also known as DP1 and DP2, respectively. PGD_2_ is a key mediator in vasodilatation [3], bronchoconstriction [4], inhibition of platelet aggregation [5]–[7], artherosclerosis [8], glycogenolysis [9], allergic reaction mediation [10], inflammation [11], [12], and intraocular pressure reduction [13]. It has also been shown to be involved in regulation of sleep [14], body temperature [15], hormone release [16], and bone metabolism [17]–[20]. DP1 is coupled to Gα_s_ and its activation by PGD_2_ leads to an increase in intracellular cAMP. Conversely, DP2 is coupled to Gα_i/o_ which results in the inhibition of adenylyl cyclase and the increase of intracellular calcium [21,21].

Several DP1 and DP2 ligands have been described [22], [23]. While PGD_2_ activates both DP1 and DP2, BW245C has been characterized as a specific DP1 agonist [24], [25]. Both of these agonists were reported to be blocked in their activation of adenylate cyclase by the antagonist BWA868C (a hydantoin compound structurally related to BW245C) in rabbit non-pigmented ciliary epithelial cells [26]. MK-0524, an indole-based acetic acid derivative, is a potent, selective DP1 antagonist that inhibits PGD_2_-induced accumulation of cAMP in both washed platelets and platelet-rich plasma with IC_50_ values of 0.09 and 4.0 nM, respectively [27]. MK-0524, also known as laropiprant, has proven to be effective in suppressing flushing symptoms due to vasodilatation with associated discomfort in humans taking nicotinic acid, commonly used to treat dyslipidemia [28].

In the last decade it has been recognised that a single receptor can engage different signaling pathways and that various ligands binding to this receptor can differentially affect each of these pathways. For instance, ligands that behave as agonists toward a given pathway can act, through the same receptor, as antagonists or inverse agonists on a different pathway in the same cell. These observations were variously referred to as biased agonism, ligand-biased efficacy, collateral efficacy, or functional selectivity [29]. For example, ICI118,551 and propranolol, which act as inverse agonists on the β_2_-adrenergic receptor toward the adenylyl cyclase signaling pathway, were shown to be partial agonists when tested on the extracellular signal-regulated kinase (ERK) activity [30]. Similar dual activities were reported for ligands acting on the H3-histamine receptor [31], the δ-opioid receptor [32], the serotonin 5-HT_2c_ receptor [33] and the dopamine D_2_L receptor [34] among others.

The concept of constitutively active GPCRs is now firmly rooted in receptor pharmacology [35]. The notion of constitutive activity refers to the ability of a receptor to produce a response in the absence of an agonist. This necessitated a revised ligand classification, and a new category of inverse agonists was introduced alongside agonist and neutral antagonist ligands. Accordingly, many molecules classified as GPCR antagonists revealed to be inverse agonists. In a multiple-state model of receptor activation whereby a receptor may exist in active or inactive conformations, an inverse agonist is a ligand that binds with higher affinity to inactive receptors over active ones [36], [37]. Equally, it is a ligand that, when bound to receptor, decreases the propensity for receptor activation [35].

New notions also emerged about previously reported antagonists now described as pharmacochaperones that rescue folding, trafficking and function of receptors [38], [39]. This has been demonstrated with several mutants of the V2 vasopressin receptor that were rescued by vasopressin antagonists [40]. Such pharmacological chaperone activity has been discovered for GPCRs involved in conformational diseases, as exemplified by the small nonpeptidic GnRH antagonists on the functional rescue of the GnRH receptor [41].

In light of the concepts of inverse agonism and pharmacochaperones, data that we obtained using MK-0524 prompted us to further characterize this molecule on DP1 signaling and cell surface expression. In the present study, we show that MK-0524 is an inverse agonist of DP1, decreasing its cAMP signaling below basal levels with no detectable effect on ERK1/2 activation. We also demonstrate that MK-0524 acts as a pharmacochaperone to favor DP1 cell surface expression.

## Materials and Methods

### Reagents

Monoclonal anti-FLAG (M2) (cat. F3165), monoclonal anti-FLAG (M1) (cat. F3040), and goat alkaline phosphatase-conjugated anti-mouse IgG (cat. A3562) antibodies were from Sigma-Aldrich, MO. Monoclonal HA.11 antibody (MMS-101R) was from Babco, CA. Calnexin polyclonal antibody (cat. SPA-865) was from Stressgen, MI. Alexa Fluor 488 donkey anti-mouse (cat. A-21202) and Alexa Fluor 546 goat anti-rabbit (cat. A-11035) secondary antibodies were from Molecular Probes, CA. Phospho-p44/p42 MAPK (Erk1/2) (Thr202/Tyr204) and total p44/42 MAPK (Erk1/2) antibodies were from Cell Signaling Technology (Danvers, Ma). Sheep peroxidase-conjugated anti-mouse IgG (NA931) and donkey anti-rabbit peroxidase-conjugated IgG antibodies were from Amersham pharmacia biotech, NJ. PGD_2_, BW245C, and BW368C were purchased from Cayman Chemical (Ann Arbor, MI) while MK-0524 was obtained from Axon Medchem (cat. Axon1480), The Netherlands. Brefeldin A was purchased from Cayman Chemical (Ann Arbor, MI).

## Plasmid Constructs

The pcDNA3-Flag-DP1, pcDNA3-dynamin-K44A and pcDNA3-Flag-TPβ constructs were described previously [42], [43].

### Cell culture, Transfection and Stimulation **−**

HEK293 cells were maintained in Dulbecco's modified Eagle's medium (DMEM) supplemented with 10% fetal bovine serum (Invitrogen) at 37°C in a 5% CO_2_ humidified atmosphere. Transfections were performed at 50–70% confluence using *Trans*IT-LT1 Reagent (Mirus, Madison, WI, USA) according to the manufacturer's protocol. Empty pcDNA3 vector was added to keep the total DNA amount added per plate constant. Whenever agonist or antagonist treatments were required, the culture medium was changed to medium containing the relevant compound (DMEM 1% BSA, 20 mM Hepes) for different times of incubation, as indicated in each experiment.

### Receptor Cell Surface Expression and Internalization Assays

ELISA were performed for quantification of receptor cell surface expression and internalization. HEK293 cells were plated out at 7.5×10^5^ cells/well and grown overnight in 24-well plates pre-coated with 0.1 mg/ml poly-L-lysine (Sigma). The cells were then transfected with empty pcDNA3 vector and pcDNA3-Flag-DP1 using a total of 166 ng DNA/well. Transfected cells were maintained for 24 h and then subjected to ligand stimulation in serum-free DMEM containing 20 mM Hepes and 1% Bovine serum albumin (BSA) for 0 to 24 h at a final concentration of 1 µM as indicated. After another 24 h, the reactions were stopped by removing the medium and fixing the cells in 3.7% formaldehyde/TBS (20 mM Tris pH 7.5, 150 mM NaCl) for 5 min at room temperature. The cells were then washed three times with TBS and nonspecific binding blocked with TBS containing 1% BSA for 45 min at room temperature. The first antibody was added at a dilution of 1∶1000 in TBS/BSA for 1 h at room temperature. Three washes with TBS followed, and cells were briefly re-blocked for 15 min at room temperature. Incubation with a goat alkaline phosphatase-conjugated anti-mouse antibody (Bio-Rad) diluted 1∶1000 in TBS/BSA was carried out for 1 h at room temperature. The cells were washed three times with TBS and a colorimetric alkaline phosphatase substrate was added (Bio-Rad). The reactions were stopped after 45 min and 100-µl samples were taken for colorimetric readings. Cells transfected with pcDNA3 were studied concurrently to determine background. All experiments were done in triplicate.

### Confocal Microscopy

HEK293 cells were plated and transfected as described above, before being transferred onto coverslips coated with 0,1 mg/mL poly-L-lysine (Sigma). Cells were then starved for 30 min and stimulated overnight with 1 µM MK-0524 or vehicle. Cells were fixed with 3% paraformaldehyde in phosphate-buffered saline (PBS) for 10 min at room temperature, washed with PBS, permeabilized with 0.1% Triton X-100 in PBS for 20 min, and blocked for 30 min with 0.1% Triton X-100 in PBS containing 5% nonfat dry milk. Cells were then incubated with primary antibodies diluted in blocking solution for 60 min, washed twice with PBS, blocked again with 0.1% Triton X-100 in PBS containing 5% nonfat dry milk for 30 min, and incubated with appropriate secondary antibodies diluted in blocking solution for 60 min. The cells were washed thrice with PBS and the coverslips finally mounted using ProLong Gold antifade reagent (cat. P36934, Molecular Probes, CA). Five-layers Z-stack acquisitions were performed using the 63x oil immersion objective of a FV1000 confocal microscope (Olympus, Japan). Acquisitions were performed sequentially for each channel and all images processed using the FV10-ASW 2.0.1.0 viewer software (Olympus, Japan).

### Total Cell Expression of Receptor Protein

To determine the total expression of receptors with or without ligands, HEK293 cells were plated at 8×10^5^ cells/60-mm plate and transfected the day after with pcDNA3-Flag-DP1. On day 3, cells were treated for 24 h with 1 µM MK-0524 or ethanol (vehicle). Cells were then washed with PBS and harvested in 300 µl of lysis buffer (150 mM NaCl, 50 mM Tris, 1% IGEPAL, 0.5% sodium deoxycholate, 10 mM Na_4_PP, 0.1% SDS, 5 mM ethylenediaminetetraacetic acid (EDTA) pH 8.0 supplemented with protease inhibitors 9 nM pepstatin, 9 nM antipain, 10 nM leupeptin and 10 nM chymostatin (Sigma)). Following incubation at 4°C for 60 min, the lysates were clarified by centrifugation for 20 min at 14 000×g at 4°C. Samples of 25 µl were analysed by SDS-PAGE and immunoblotting using specific antibodies.

### Brefeldin A on MK0524 Pharmacochaperone Activity

Cells prepared as above for assessment of DP1 cell surface expression by ELISA were pretreated for 30 min with 20 µM Brefeldin A (BFA) and then incubated with 1 µM of MK-0524 for 90 min. The reactions were stopped by removal of media and treatment with formaldehyde 3.7% for 5 min. Samples were analysed by ELISA.

### Intracellular cAMP Measurements

cAMP accumulation was determined using a commercial cAMP enzyme immunoassay Biotrak (EIA) system (RPN225, GE Healthcare) according to the manufacturer’s instructions. HEK293 cells were plated into 6-well plates at a density of 2.9×10^5^ cells/well. They were transfected 24 h later with pcDNA3-Flag-DP1 and pcDNA3 as indicated. The following day, transfected cells were manually detached and transferred at a density of 3×10^4^ cells/well into 96-well plates pre-coated with 0.1 mg/ml poly(L)-lysine and incubated overnight. Cells were pre-incubated with IBMX (3-isobutyl-1-methylxanthine), and with 1 µg/ml pertussis toxin (P7208) in selected experiments, for 10 min and then stimulated with the indicated drugs for another 10 min at the indicated concentrations. The non-acetylated cAMP generated under the different conditions was interpolated from a cAMP standard curve generated in parallel for each experiment. Triplicates were used for each condition, and all experiments were repeated at least three times.

### ERK1/2 Phosphorylation

HEK293 cells were seeded in poly-(L)-lysine-coated 6-well plates at 2,4×10^5^ cells per well and grown for 24 h before being transfected with the indicated constructs. Forty-eight hours post-transfection, cells were starved for 30 min in DMEM supplied with 0.5% BSA and 20 mM Hepes pH 7.5 and then treated with 1 µM PGD_2_ or MK-0524 for the indicated times. The reactions were stopped with 350 µl of 1x sample buffer (62.5 mM Tris pH 7.0, 2% w/v SDS, 10% glycerol, 50 mM DTT, 0.01% w/v bromophenol blue) and sonicated. The samples were then analyzed by Western blotting using phospho-p42/p44 and secondary horseradish peroxidase-conjuted anti-rabbit antibodies. Blots were then stripped and re-probed with p42/p44 antibodies. Experiments were done at least 3 times for each ligand.

### Immunoprecipitation of DP1

HEK293 cells were transiently transfected with the indicated constructs and were maintained as described above for 48 h. Where indicated, cells were incubated in the presence of 1 µM of MK-0524 for 24 h before harvesting. The cells were then washed with ice-cold PBS and harvested in 200 µl of lysis buffer (150 mM NaCl, 50 mM Tris (pH 8.0), 0.5% deoxycholate, 0.1% SDS, 10 mM Na_4_P_2_O_7_, 1% IGEPAL, and 5 mM EDTA or 1 mM CaCl_2_ depending on the antibody used for the assay) supplemented with protease inhibitors (9 nM pepstatin, 9 nM antipain, 10 nM leupeptin and 10 nM chymostatin) (Sigma-Aldrich). After 45 min of incubation in lysis buffer at 4°C, the lysates were centrifuged for 15 min at 14,000×g at 4°C. Flag-DP1 was immunoprecipitated for 60 min using 1 µg of specific antibodies before adding 40 µl of 50% protein G-agarose beads to the lysates for 30 min. Samples were then centrifuged for 2 min in a microcentrifuge and washed three times with 1 ml of lysis buffer. Immunoprecipitated proteins were eluted by addition of 35 µl of SDS sample buffer, followed by an incubation of 60 min at room temperature. Initial lysates and immunoprecipitated proteins were analyzed by SDS-PAGE and immunoblotting with specific antibodies.

### Statistical Analysis

Statistical analyses were performed using Prism v5.0 (GraphPad Software, San Diego, CA, USA) using the Student’s t-test. Data were considered significant when *P* values were <0.05 (*), 0.01 (**), or 0.001(***).

## Results

### MK-0524 is an Inverse Agonist of DP1 cAMP Signaling

**Figure 1:**
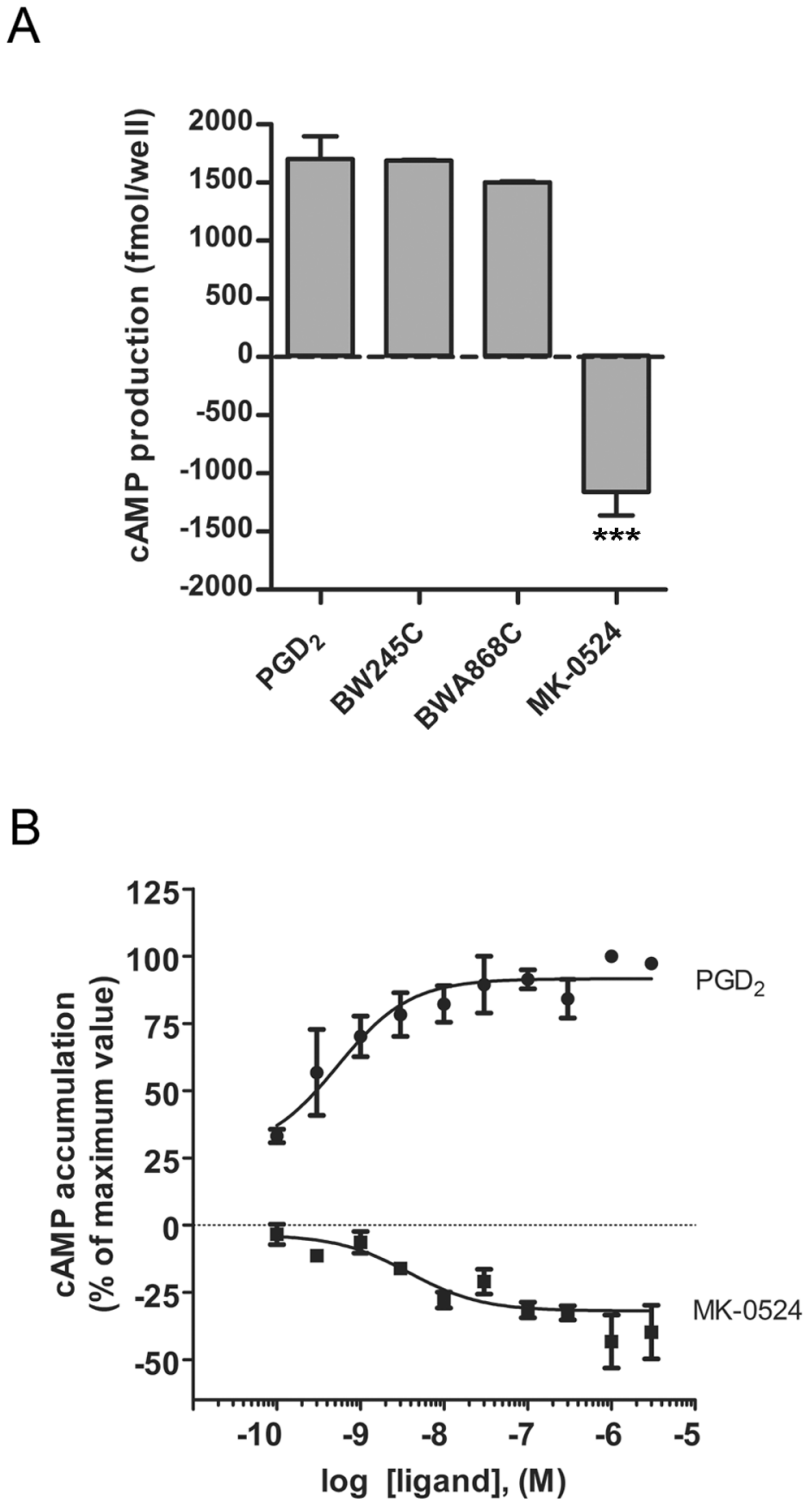
MK-0524 reduces DP1 cAMP signaling below basal levels. HEK293 cells transiently expressing Flag-DP1 were stimulated with 10 nM (A) or increasing concentrations (B) of the indicated ligands for 10 min and cAMP levels were measured as described in “Materials and Methods”. Results are presented in fmol/well above or below basal cAMP production by DP1 in absence of ligand (set at 0) (A) or as the % of the maximal response obtained with PGD_2_ stimulation (B). Data are the mean ± S.E. of at least three independent experiments. *** is *P*<0.001.

The ability of BWA868C and MK-0524 to block PGD_2_-induced cAMP generation has already been characterized [25], [27], [44], [45]. Here, we were interested in comparing the effects of the individual molecules on DP1-mediated cAMP signaling in DP1-expressing HEK293 cells following treatment with 10 nM of the DP1 agonists PGD_2_ and BW245C, or the previously characterized antagonists BWA868C and MK-0524. PGD_2_ and BW245C generated virtually identical responses in cAMP generation (Fig. 1A). BWA868C treatment also resulted in cAMP production, which corresponded to 88% of the PGD_2_ response, in accordance with its partial agonist properties reported by other groups [7], [46]–[48]. In contrast, incubation with MK-0524 resulted in a decrease in cAMP generation below DP1 basal activity (Fig. 1A). Concentration-response experiments of cAMP production were then generated to further characterize this novel signaling property for MK-0524 (Fig. 1B). The decrease in DP1 cAMP signaling mediated by MK-0524 reached a plateau at ∼300 nM with an EC_50_ of 3.8±0.3 nM.

**Figure 2:**
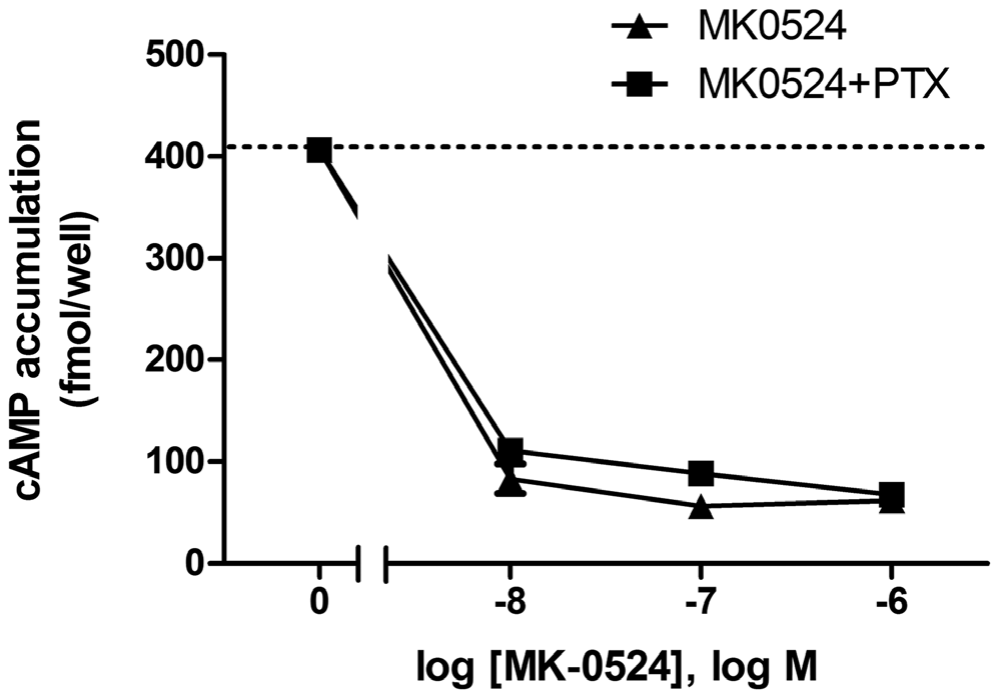
The MK-0524-mediated reduction in DP1 cAMP signaling below basal levels is unaffected by pertussis toxin. HEK293 cells transiently expressing Flag-DP1 pretreated or not with 1 µg/ml of pertussis toxin (PTX) for 10 min were incubated with increasing concentrations of MK-0524 and cAMP levels were measured as described in “Materials and Methods”. Results are presented as fmol of cAMP generated per well. Data are the mean ± S.E. of at least three independent experiments.

Diminished cAMP generation can be caused by Gα_i/o_-coupling of a receptor. Although DP1 is known to be coupled to Gα_s_, we still wanted to assess whether MK-0524 reduced cAMP levels by DP1 through Gα_i/o_ signaling in the context of functional selectivity. Cells were thus treated with increasing concentrations of MK-0524 in presence of pertussis toxin (PTX) to inhibit Gα_i/o_ signaling [11] or with control vehicle. Fig. 2 shows that the dose-response curve of cAMP reduction below basal levels by treating DP1-expressing cells with MK-0524 was not affected by PTX. These data thus demonstrate that MK-0524 acts as an inverse agonist for DP1 cAMP generation. To our knowledge, this is the first description of an inverse agonist for DP1.

**Figure 3:**
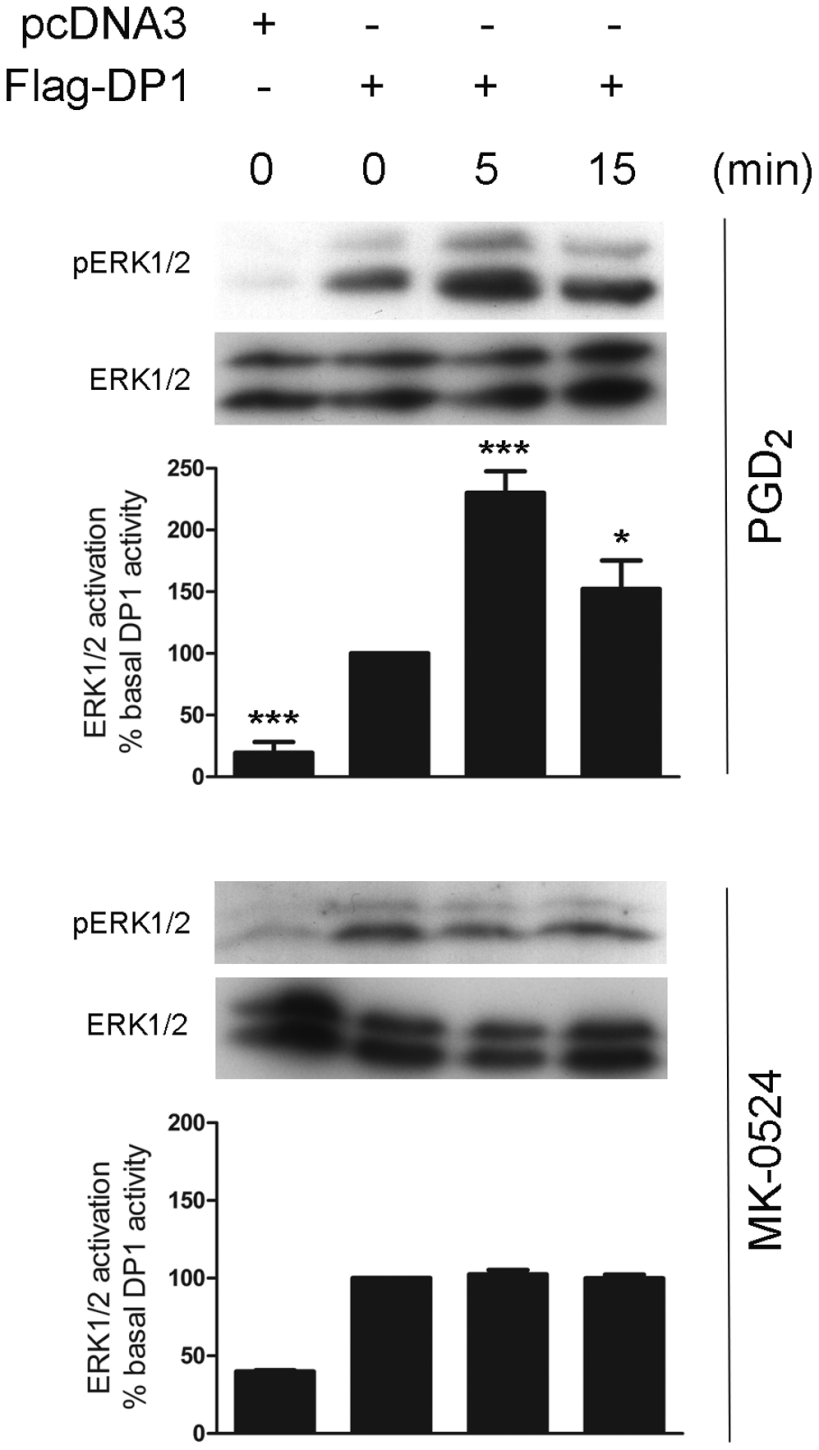
MK-0524 does not modulate ERK1/2 activation by DP1. HEK293 cells transiently expressing Flag-DP1 were stimulated with 1 µM PGD_2_ or MK-0524 for the indicated times. ERK1/2 activation was analyzed by Western blot using a phospho-ERK1/2 (pERK1/2) antibody as described under “Materials and Methods”. Total amounts of ERK1/2 in the loaded samples were revealed by an anti-ERK1/2 antibody. The blots shown are representative of three separate experiments. Densitometry analyses (pERK1/2/ERK) of at least three different experiments were performed. *IB*, immunoblotting. * is *P*<0.05 and *** is *P*<0.001.

We next studied the effect of MK-0524 on another DP1 signaling pathway. DP1-expressing HEK293 cells were stimulated with PGD_2_ or MK-0524 and ERK1/2 activation was measured by Western blotting using a phospho-ERK1/2 antibody. Significant basal ERK1/2 activation was observed when DP1 was expressed in absence of ligand stimulation (time 0) compared to cells transfected with pcDNA3 (Fig. 3). ERK1/2 activation by PGD_2_ was detected after 5 min of DP1 stimulation (Fig. 3). On the other hand, MK-0524 had no noticeable effect on ERK1/2 activation by DP1-expressing cells (Fig. 3). Our data show that MK-0524 is an inverse agonist for cAMP signaling by DP1, but has no detectable effect on ERK1/2 activation.

### MK-0524 Acts as a Pharmacochaperone in Promoting DP1 Cell Surface Expression

**Figure 4:**
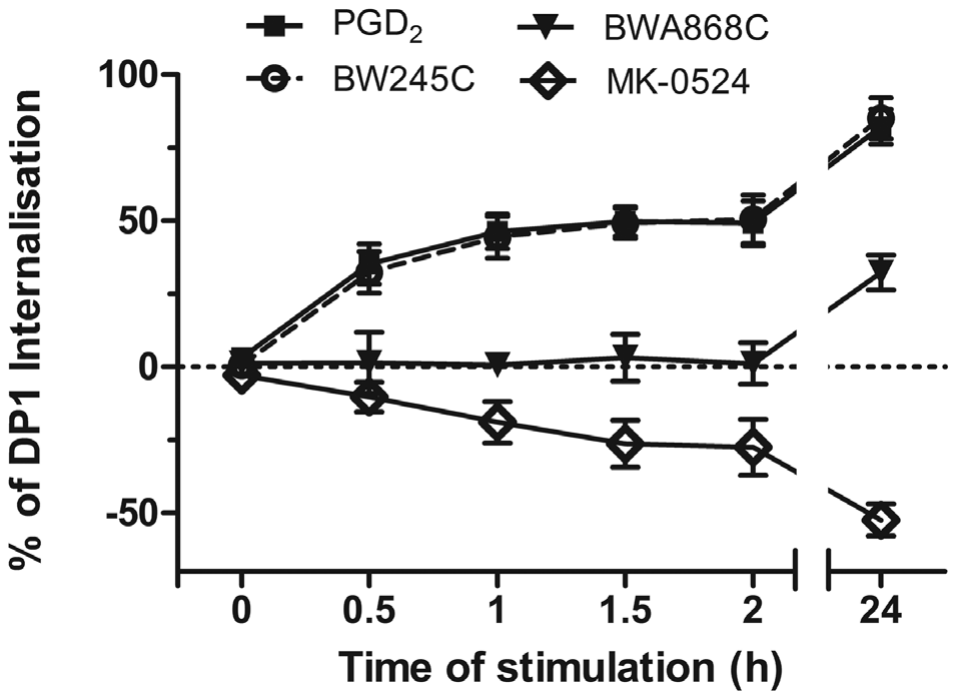
MK-0524 promotes cell surface expression of DP1. Cell surface expression of the receptor was measured by ELISA as described under “Materials and Methods” in HEK293 cells transiently expressing Flag-DP1 incubated for the indicated times with 1 µM of PGD_2_, BW245C, BWA868C or MK-0524. The results are shown as the percentage of cell surface expression of Flag-DP1 in cells stimulated with the ligands compared with cells treated with control vehicles. Results are the mean ± S.E. of at least four independent experiments.

We were also interested in comparing the effects of PGD_2_, BW245C, BWA868C and MK-0524 on DP1 cell surface expression. HEK293 cells expressing FLAG-tagged DP1 were subjected to time-course stimulations with the different DP1 ligands. Quantification of DP1 at the cell surface was performed by ELISA (Fig. 4), as we did before [42], [43], [49], [50]. Stimulation of cells with PGD_2_ and BW245C resulted in identical DP1 internalization curves that reached a plateau after 1 h of agonist stimulation where roughly 50% of receptors were lost from the cell surface. Treatment with either agonist for 24 h induced internalization of 75% of DP1. In contrast to its important partial agonist activity in cAMP generation assays, BWA868C did not significantly affect DP1 cell surface expression for the first 2 h, but promoted internalization of 25% of DP1 after 24 h of treatment. Remarkably, MK-0524 increased DP1 cell surface expression time-dependently by 25% and 50% after 2 h and 24 h of incubation, respectively.

**Figure 5:**
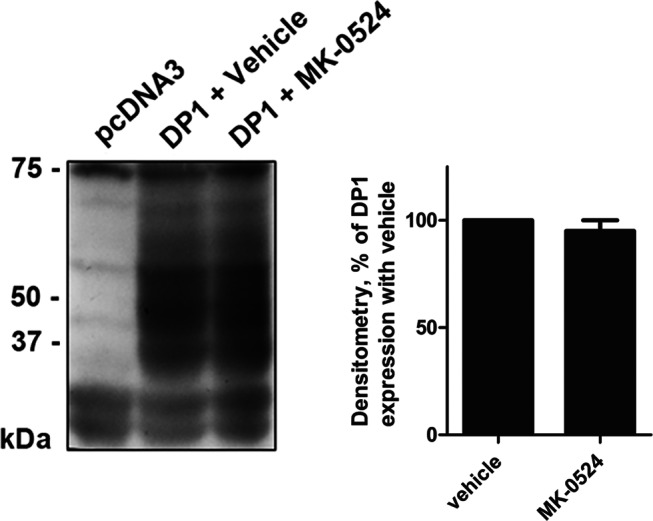
The total expression of the DP1 protein is not modulated by MK-0524. Lysates of HEK293 cells transiently expressing Flag-DP1 incubated for 24 h with vehicle or 1 µM of MK-0524 were analyzed by Western blot using a monoclonal Flag antibody. The blot shown is representative of three separate experiments. *IB*, immunoblotting.

**Figure 6:**
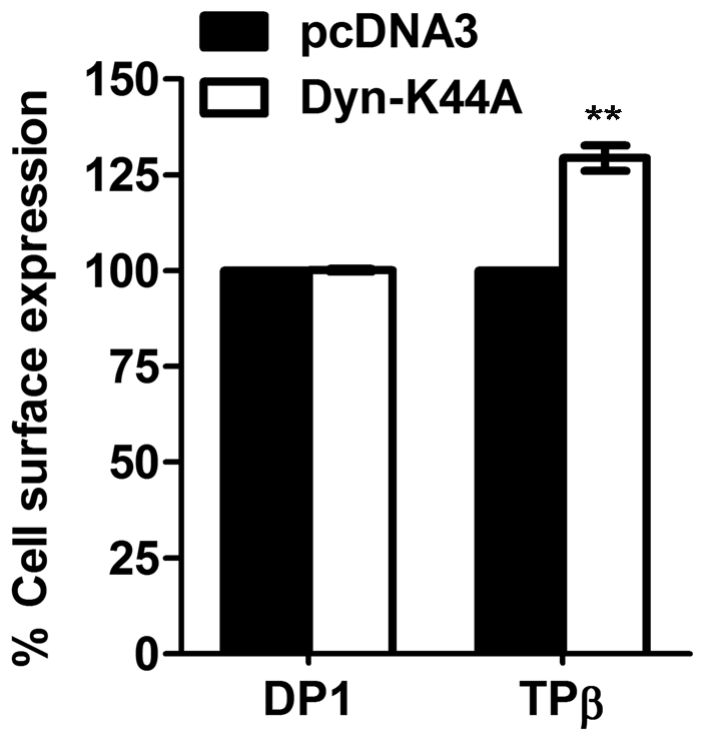
DP1 does not undergo constitutive internalization. Cell surface receptor expression was measured by ELISA as described under “Materials and Methods” in HEK293 transiently co-transfected with pcDNA3-Flag-DP1 or pcDNA3-Flag-TPβ in combination with pcDNA3 or pcDNA3-dynamin-K44A, a dominant negative mutant of dynamin. Data are shown as the percentage of cell surface receptor expression in cells co-transfected with pcDNA3 for each receptor. Results are the mean ± S.E. of at least three independent experiments. ** is *P*<0.01.

Western blot analysis revealed that MK-0524 did not modulate the levels of total DP1 protein expression (Fig. 5). This indicated that enhanced DP1 cell surface expression was not caused by increased receptor protein levels following treatment with MK-0524. Many GPCRs undergo constitutive internalization [43], [51]–[54]. Inhibition of tonic/constitutive internalization of DP1, or prevention of the formation of active states of the receptor that would normally internalize, by MK-0524 would result in the accumulation of receptors at the cell surface [43], [51], [52] which could be interpreted as increased cell surface targeting of receptors. We previously reported that agonist-induced internalization of DP1 was inhibited by a dominant-negative mutant of dynamin, dyn-K44A [43]. To investigate whether DP1 undergoes constitutive internalization, we measured cell surface expression of the receptor in HEK293 cells expressing Flag-DP1 alone or in combination with dyn-K44A, which also blocks constitutive internalization [29], [51], [52]. Our data show that DP1 is not subjected to constitutive internalization, as opposed to the Flag-TPβ receptor (Fig. 6), as we previously reported for both receptors [43], [52]. This demonstrates that the increase in DP1 cell surface expression by MK-0524 cannot be due to inhibition of constitutive internalization of the receptor.

**Figure 7:**
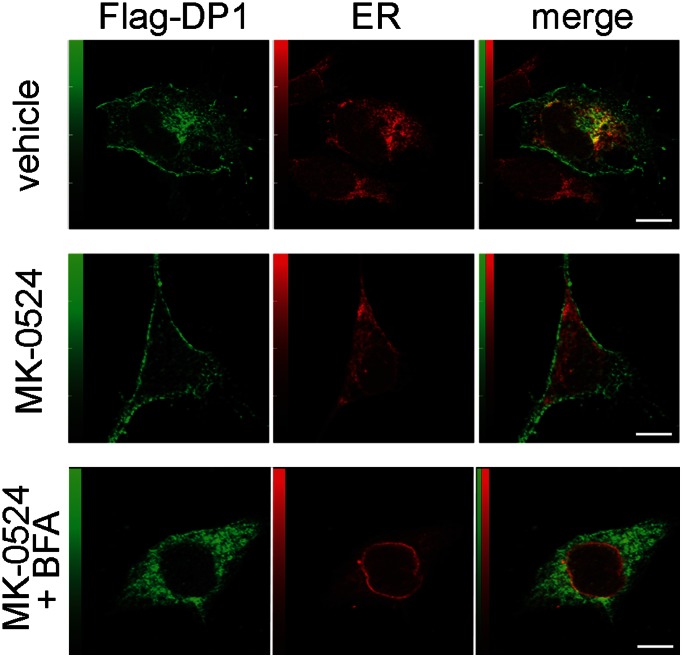
MK-0524 induces translocation of DP1 from intracellular compartments to the plasma membrane. The distribution of DP1 in HEK293 cells was determined by immunofluorescence confocal microscopy. HEK293 cells transfected with Flag-DP1 were treated with vehicle or 1 µM of MK-0524 alone or in presence of 20 µM Brefeldin A for 90 min. Cells were labeled with mouse anti-FLAG and either a rabbit anti-calnexin antibody (top and midlle panels) or a rabbit anti-protein disulfide isomerase (PDI) antibody (lower panel) as described under “Materials and Methods”. Secondary antibodies were Alexa Fluor 488 donkey anti-mouse IgG and Alexa Fluor 546 goat anti-rabbit IgG. Merge images of the green-labelled DP1 and red-labeled calnexin or PDI are shown. Bars, 10 µM.

During prior studies, we observed that a significant population of DP1 was retained intracellularly [42]. We thus hypothesized that MK-0524 could favor the transport of DP1 from intracellular compartments to the plasma membrane. To test our hypothesis, we performed immunofluorescence confocal microscopy on HEK293 cells expressing FLAG-DP1 that were treated with vehicle or MK-0524 (Fig. 7). In cells treated with the vehicle, DP1 was localized at the plasma membrane and in a considerable proportion in intracellular compartments. Significant DP1 co-localization was detected with calnexin, an endoplasmic reticulum marker (Fig. 7, upper panel). In contrast, treatment of cells with MK-0524 for 24 h resulted in detection of DP1 mostly at the plasma membrane with very little intracellular receptor immunofluorescence remaining and no detectable receptor co-localization with calnexin (Fig. 7, middle panel). Treatment of cells with 20 µM Brefeldin A, an inhibitor of transport from the ER-Golgi to the plasma membrane, prevented the MK-0524-induced redistribution of the intracellular receptors to the plasma membrane (Fig. 7, lower panel).

**Figure 8:**
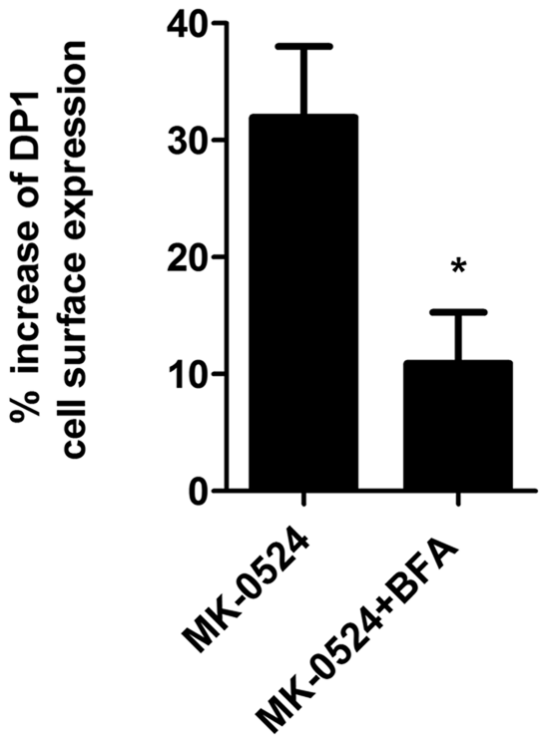
The promotion of DP1 cell surface targeting by MK-0524 is inhibited by Brefeldin A. Cell surface receptor expression was measured by ELISA as described under “Materials and Methods” in HEK293 cells transiently expressing Flag-DP1 that were pre-incubated with vehicle or 20 µM of Brefeldin A (BFA) for 30 min, and then treated with 1 µM of MK-0524 or its control vehicle for 90 min. The results are shown as the percentage increase of DP1 cell surface expression when compared to control cells treated with vehicle. Results are the mean ± S.E. of three independent experiments. * is *P*<0.05.

To confirm these data, DP1 cell surface expression was measured by ELISA in DP1-expressing HEK293 cells that were pretreated with 20 µM Brefeldin A and then subjected to stimulation with MK-0524 for 90 min. Fig. 8 shows that the MK-524 treatment alone resulted in a 32% increase in DP1 cell surface expression, whereas this response was down to 11% in cells pretreated with Brefeldin A. Together, these results indicate that MK-0524 acts as a pharmacochaperone in promoting the redistribution of DP1 from intracellular compartments to the plasma membrane.

**Figure 9:**
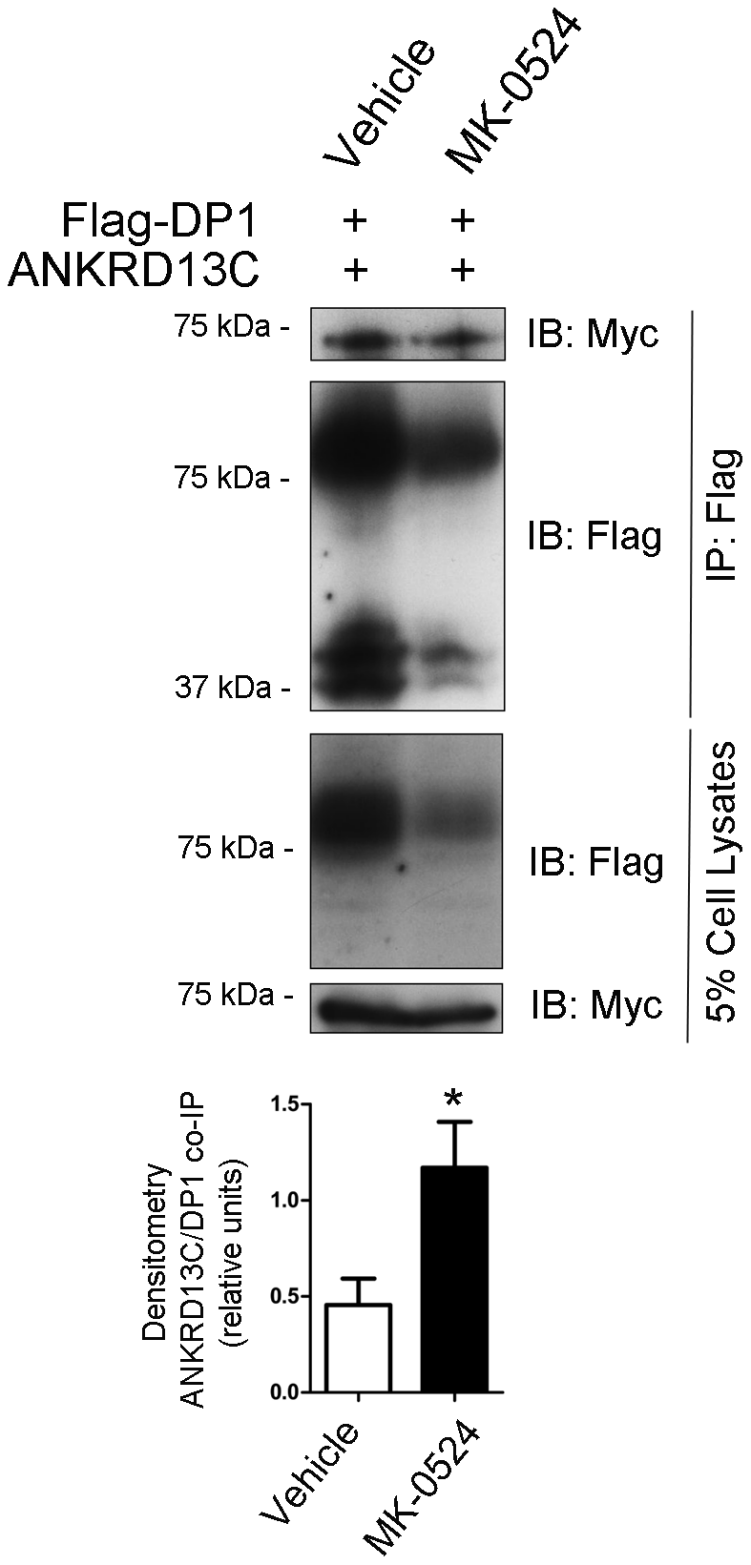
MK-0524 promotes the interaction between DP1 and the ANKRD13C protein. HEK293 cells transiently co-expressing Flag-DP1 and ANKRD13C-myc were treated with vehicle (ethanol) or 1 µM MK-0524 for 24 h. Flag-DP1 was immunoprecipitated as described under “Materials and Methods” and immunoprecipitated samples as well as cell lysates were analyzed by Western blot with anti-Flag and anti-Myc antibodies. The blots shown are representative of three separate experiments. The ratio of the amount of ANKRD13C that was co-immunoprecipitated on the quantity of receptor immunoprecipitated was calculated by densitometry analyses from the three separate experiments and the results are shown in the bottom panel as the mean ± S.E. * is *P*<0.05.

To further support the notion that MK-0524 is acting as a DP1 pharmacochaperone, we evaluated its capacity to modulate the interaction between DP1 and various molecular chaperones involved in quality control of GPCRs. Unfortunately, immunoprecipitation experiments of overexpressed DP1 in HEK293 cells failed to reveal interactions between the receptor and chaperones like BiP, calnexin, calreticulin, Hsc70 and Hsp90 (data not shown). However, we recently reported that ANKRD13C displayed chaperone-like properties towards DP1 at the ER-Golgi level [42]. Prolonged interaction between ANKRD13C and DP1 targeted the receptor for proteasomal degradation. The DP1-ANKRD13C interaction was not modulated by PGD_2_ stimulation [42]. Interestingly, Fig. 9 shows that incubation with MK-0524 for 24 h of Flag-DP1 overexpressing cells transfected with ANKRD13C promoted the DP1-ANKRD13C interaction. Indeed, a similar amount of ANKRD13C was co-immunoprecipitated even though much less receptor was immunoprecipitated and densitometry analysis confirmed the increased interaction between the two proteins in presence of MK-0524. In these conditions, total DP1 protein expression was decreased after incubation with MK-0524 which makes sense given that the molecule promotes the interaction between the receptor and ANKRD13C, which targets immature/unfolded DP1 to degradation [42]. This indicates that MK-0524 can modulate the association between DP1 and proteins involved in its quality control.

## Discussion

It has become apparent in the last twenty years or so that the original model of GPCRs existing as “on” and “off” is overly simplified. Many ligands were discovered to display biased agonism or functional selectivity [29], [55]–[61]. Functional selectivity describes the capacity of ligands to modulate selectively one or several pathways among all those that can be activated by a receptor [29]. A major advantage of functional selectivity is that it allows to develop new and smarter drugs that selectively affect GPCR signaling responsible for a desired therapeutic effect while causing no or lesser activity on signaling pathways responsible for adverse effects [55], [56]. Another crucial development in our understanding of GPCR function has been an appreciation of their ability to activate cognate G proteins in the absence of agonist binding, termed “constitutive receptor signaling” [62], [63]. This was followed by the identification of ligands named “inverse agonists”. Inverse agonism is the property of a ligand to produce a decrease in the basal level of signaling after binding to a receptor [64]. Multiple ligands that were initially characterized as antagonists have proven to be inverse agonists [64]–[68].

BWA868C and MK-0524 were described as PGD_2_ antagonists of DP1-mediated cAMP generation in platelets [27], [45], [68] and HEK293 cells [1]. Data that we obtained in previous control experiments (not shown) prompted us to further characterize the effects of these two DP1 antagonists individually on the receptor. PGD_2_ induced cAMP generation with an EC_50_ that was consistent with what was described in endogenous tissues and in HEK293 cells [1], [24], [46], confirming the suitability of our system. When added alone at 100 nM, BWA868C induced cAMP generation at lower levels than PGD_2_, in agreement with observations made by other groups [7], [46], [48]. Interestingly, MK-0524 reduced basal levels of cAMP production by DP1 below its basal levels. DP1 is known to be a Gα_s_-coupled receptor. Given that many receptors can couple to more than one G protein, it is increasingly accepted that the diverse ligand-promoted receptor conformations can yield differential signaling efficacies through distinct effector systems. In this context, we thus tested whether MK-0524 decreased DP1-mediated cAMP generation through Gα_i/o_ signaling by treating cells with pertussis toxin. The reduction in cAMP generation below basal levels by MK-0524 in DP1-expressing cells was not affected by pertussis toxin treatment, showing that this was not due to coupling of DP1 to Gα_i/o_. Furthermore, HEK293 cells do not produce PGD_2_ [69] so the MK-0524-mediated decrease in DP1 basal cAMP signaling was not caused by displacement of endogenous PGD_2_. If the latter situation was occurring, one would expect MK-0524 to also decrease DP1 basal ERK1/2 activation, which is not the case. It is interesting to note that expression of DP1 in absence of agonist resulted in significant basal ERK1/2 activation compared to cells transfected with pcDNA3. The fact that MK-0524 does not reduce this basal ERK1/2 activation might suggest that it is an inverse agonist specific to DP1 cAMP signaling. Very few biased inverse agonists of GPCRs have been described so far [70]–[72]. Alternatively, the lack of an observable effect of MK-0524 on basal DP1 ERK1/2 activation could be explained by limitations in the sensitivity of the assay or low potency of the ligand in this signaling pathway. We thus report that the DP1 specific antagonist MK-0524 is an inverse agonist of DP1 towards cAMP signaling in our system. To our knowledge, this is the first description of an inverse agonist for DP1.

The effects of PGD_2_, BW245C, BWA868C and MK-0524 on DP1 cell surface expression were also studied. As we reported before [19], both DP1 agonists PGD_2_ and BW245C induced internalization of 50% and 75% of the receptors after 2 h and 24 h of stimulation, respectively. Interestingly, BWA868C, which displayed significant agonist activity in DP1-mediated cAMP generation, did not induce DP1 internalization during the first 2 h of receptor stimulation, but induced internalization of 25% of the receptors after 24 h of treatment. In contrast to the other ligands used in the present study, treatment with MK-0524 promoted DP1 cell surface expression by 50% over a 24 h period. According to different reports, it has become evident that many GPCRs exhibit a poor rate of trafficking to the cell surface leading to restricted plasma membrane expression [39], [73]–[83]. We recently observed that a significant proportion of DP1 was localized in intracellular compartments [42]. This intracellular retention could be a system developed by the cell to control the number of receptors at the plasma membrane. In the last decade, it has been found that translocation of intracellularly retained receptors to the plasma membrane could be rescued by drugs that act as pharmacochaperones [73]. The results presented in this report indicate that MK-0524 can act as a pharmacochaperone for DP1 in HEK293 cells. Confocal microscopy analysis confirmed that a significant proportion of DP1 was localized intracellularly and co-localized with the endoplasmic reticulum marker calnexin in cells treated with control vehicle. Incubation with MK-0524 resulted in translocation of DP1 from intracellular compartments to the plasma membrane with very little DP1 remaining in intracellular compartments. This translocation was inhibited by disruption of ER/Golgi transport to the plasma membrane with Brefeldin A as evidenced visually by confocal microscopy and quantitatively by DP1 cell surface expression assays. Increase in DP1 cell surface expression by MK-0524 could have been the result of inhibition of tonic/constitutive internalization of the receptor, like we showed to occur for TPβ, or prevention of spontaneously formed active states of the receptor that normally would internalize. Experiments with dynamin-K44A showed that the MK-0524-mediated increase in DP1 cell surface expression could not be due to inhibition of constitutive internalization of the receptor. Furthermore, MK-0524 promoted the interaction between DP1 and ANKRD13C, which we showed to have chaperone-like effects in determining the fate of DP1 [42]. Similarly to the quality control chaperone machinery, ANKRD13C can promote DP1 expression but can also target immature/unfolded DP1 to proteasomal degradation [42]. The increased DP1-ANKRD13C interaction by MK-0524 could prevent accumulation of misfolded forms of DP1 in the ER that would result in aggregate formation and intracellular retention of the receptors. It is interesting that MK-0524, but not PGD_2_, modulates the DP1-ANKRD13C interaction. This could reflect different abilities of the ligands to access the receptor in various cellular locations or to induce different receptor conformations that would determine interactions between the receptor and different sets of proteins. Future experiments will reveal and compare the complement of proteins that interact with DP1 in presence of a variety of ligands. Altogether, our data support the idea that MK-0524 is a pharmacochaperone of DP1 that favors transport of the receptor from the ER/Golgi to the plasma membrane.

The first demonstration that pharmacologically selective agents could rescue cell surface expression and function of GPCRs, which were otherwise retained in the endoplasmic reticulum, came from work conducted on V2 vasopressin receptor mutants responsible for nephrogenic diabetes insipidus [41], [76], [84]. Pharmacochaperones were also identified for mutants of rhodopsin and of the α_1b_-adrenergic, gonadotropin-releasing hormone and calcium-sensing receptors [41], [85]–[88]. While pharmacochaperones have been described mostly in the context of mutant GPCRs, their action has also been reported for the wild-type δ-opioid and the gonadotropin-releasing hormone receptors [41], [76], [84], and now here for DP1. The fact that wild-type GPCRs can be manipulated by pharmacochaperone treatments opens the way to the use of these molecules as regulators of tissue responsiveness in normal individuals [84]. The use of antagonists or inverse agonists as pharmacochaperones therapeutically would require a subtle balance between their ability to target the receptor to the cell surface and their possibility to be displaced by the natural ligand for receptor activation [89]. In this regard, it has been proposed that either low concentrations of pharmacochaperones with high affinity or higher concentrations of pharmacochaperones with low affinity could be used [89].

Our group has shown that PGD_2_ and DP1 are positively involved in bone matrix deposition and bone fracture repair [17], [19]. In mice, PGD_2_ was recently demonstrated to play an anti-inflammatory role in articular tissue during development of collagen-induced arthritis through DP1 [86]. In this context, it could be thought that pharmacochaperones promoting DP1 cell surface expression may have an impact on different bone and joint diseases such as osteoporosis, periodontal disease, fracture repair, rheumatoid arthritis and spondylarthropathies. The potential utility of favoring DP1 expression and activity was also proposed for treating pruritus and atopic dermatitis [90]–[93]. On the other hand, PGD_2_ is a mediator of allergic disease and favoring DP1 cell surface expression may not be desirable in this circumstance. However, the role of DP1 in regulating allergic reactions is complex [23]. For instance, the administration of BW245C in wild-type mice reduced pulmonary allergic responses whereas DP1 null mice were unaffected [23], [94]. A DP1 antagonist, S-5751, attenuated allergen-induced inflammation in sensitized guinea pigs [95]. It was recently reported that PGD_2_ signaling through DP1 between alveolar endothelial/epithelial cells and infiltrating neutrophils provides anti-inflammatory effects in acute lung inflammation, and the therapeutic potential of enhancements of PGD_2_ and DP1 signaling was suggested [96]. Thus, it is proposed that the harmful/protective actions of PGD_2_ may depend on when and where it is produced and on the PGD_2_ receptor that is activated, DP1 or DP2 [23].

MK-0524 (laropiprant) has no efficacy in patients with allergic rhinitis and asthma [90], [97]. However, laropiprant has a positive clinical outcome in limiting the cardiovascular side effects of niacin in the treatment of dyslipidemia [23]. It is plausible that in certain situations that the antagonist (inverse agonist) properties of MK-0524 are offset by its propensity to increase DP1 cell surface expression, as we described here. Careful characterization of existing and newly developed molecules targeting DP1 should be conducted to evaluate in detail their pharmacological properties in the perspective of functional selectivity and pharmacochaperone activity. DP1 antagonists or inverse agonists without pharmacochaperone activity could be useful in circumstances where DP1 activity needs to be inhibited. Conversely, in conditions where increased DP1 cell surface expression and activity is desired, for example in bone matrix deposition, a pharmacochaperone with agonist characteristics could be beneficial.

In conclusion, we have reported that MK-0524 is an inverse agonist for DP1 towards cAMP signaling and a pharmacochaperone that favors DP1 cell surface expression. These findings can be relevant to clinical applications where MK-0524 (laropiprant) is used, and to the development of new molecules targeting DP1.
